# The Role of Operation in the Treatment of Boerhaave's Syndrome

**DOI:** 10.1155/2018/8483401

**Published:** 2018-06-28

**Authors:** Dingpei Han, Zhuoqiong Huang, Jie Xiang, Hecheng Li, Junbiao Hang

**Affiliations:** ^1^Department of Thoracic Surgery, Ruijin Hospital, Shanghai Jiaotong University School of Medicine, China; ^2^Department of Respiratory, Ruijin Hospital Branch of Luwan District, Shanghai Jiaotong University School of Medicine, China

## Abstract

**Purpose:**

This study aims to discuss the appropriate treatment strategy for spontaneous esophageal rupture.

**Methods:**

Clinical data from twenty-one cases were retrospectively analyzed. The parameters included etiology, time interval between onset and treatment, therapy methods, prognosis, and length of stay.

**Results:**

The ratio of males/females was 17/4, age range was 32–82 years (mean = 43.1), and the time interval between onset and treatment was as follows: <24 h: nine cases (42.8%); 24–48 h: six cases (28.6%); and >72 h: six cases (28.6%). All patients underwent operative treatment, and the following primary healing rates were achieved: <24 h: 88.9%, 24–48 h: 66.7%, and >72 h: 0. No patients died in this study. All patients were discharged with recovery, and the average hospitalization times were 18.1 days (<24 h), 27.8 days (24–48 h), and 51.2 days (>72 h).

**Conclusions:**

Surgical treatment remains an effective method for treating spontaneous esophageal rupture, and the shorter the time interval between onset and treatment, possibly the better the prognosis.

## 1. Introduction

Spontaneous esophageal rupture was previously known as Boerhaave's syndrome, which was first described by Dr. Herman Boerhaave in 1724. It has a low incidence as one of the causes of esophageal perforation, but the rate of misdiagnosis has been reported to be as high as 50% because of its nonspecific symptoms [1]. Delayed diagnosis results in dangerous consequences such as serious mediastinal infection and sepsis, which are accompanied by high mortality. Moreover, the treatment of Boerhaave's syndrome is related to many factors, and there is as of yet no definitive treatment. In this study, we analyzed data from 21 cases of Boerhaave's syndrome over 20 years to clarify the appropriate treatment strategy.

## 2. Materials and Methods

### 2.1. Clinical Data

Clinical data from 21 patients with spontaneous esophageal rupture between January 1993 and December 2012 were collected and analyzed retrospectively. The male/female ratio was 17:4, and the mean age was 43.1 (range, 32–82) years. We excluded cases with the following criteria: traumatic esophageal rupture, tumorous esophageal rupture, foreign body esophageal rupture, and dynamic esophageal rupture. The study was approved by the ethics committee from Ruijin Hospital.

### 2.2. Parameters

We analyzed the following parameters: etiology, symptoms, time interval between onset and treatment, therapeutic methods, position and size of rupture, outcome, and length of stay.

### 2.3. Diagnosis

In the emergency department, we meticulously collected the medical history of all of the patients. After all necessary physical and auxiliary examinations were finished, we made definite diagnoses according to the following conditions: (1) Medical history and symptoms: most patients have a history of severe vomiting after consuming a large meal or an excessive amount of alcohol. Patients always suffer severe pain after vomiting, with the pain usually located at the sternum or xiphoid process, sometimes radiating to the left shoulder or abdomen. Severe cases with complications such as mediastinal infection, pleural effusion, pneumothorax, and arrhythmia have the following symptoms: high fever, shortness of breath, difficulty breathing, and shock. (2) Physical examinations: patients usually appear acutely ill. Upper abdominal tenderness or peritoneal irritation (if esophageal or gastric contents are refluxed into the abdominal cavity) can be found. Weak lung breath sounds result from pleurisy and pleural effusion. The spread of mediastinal emphysema can cause cervical subcutaneous emphysema. If the recurrent laryngeal nerves are involved, patients will possibly have voice hoarseness. Patients with serious mediastinal infection have symptoms associated with sepsis and toxic shock such as low blood pressure, thready pulse, clammy skin, and peripheral cyanosis. The following symptoms are typical of esophageal rupture and are referred to as Meckler's triad: vomiting, chest pain, and subcutaneous emphysema; however, these are only found in 30–50% of patients with esophageal rupture [2]. Most patients' symptoms are nonspecific, and differential diagnoses commonly include acute abdominal disease, myocardial infarction, and pulmonary embolism. Therefore, medical histories play an important role in the diagnosis of Boerhaave's syndrome. (3) Auxiliary examinations: chest radiography: widened mediastinum on X-ray indicates mediastinal inflammation or pneumomediastinum. Pneumothorax or hydropneumothorax results from inflammatory infiltration of the pleura. If inflammation deteriorates further, pulmonary interstitial changes can be found. In addition, some patients' X-rays show no positive performance. In diluted barium esophagography, if the contrast agent escapes to spaces in the surrounding tissues, the rupture locations are defined. Every suspicious patient with chest pain should undergo regular computed tomography (CT) examination for the early detection of esophageal pneumatosis. Endoscopic examinations can combine diagnosis and treatment, especially in case with upper gastrointestinal bleeding [3], but also require doctors with a high level of skill.

### 2.4. Therapeutic Methods

The current treatment for Boerhaave's syndrome includes surgical and conservative treatment. In this paper, the surgical treatment steps are as follows: (1) the surgical approach being decided according to the patients' symptoms, signs, and physical examination; (2) debridement; (3) whether to attempt primary repair according to the results of surgical exploration; (4) reexpanding the lungs; (5) placing a chest tube and a silicone tube to achieve adequate drainage; and (6) performing jejunostomy if necessary. It is also essential to adopt fasting, gastrointestinal decompression, washing drainage, nutritional support, and anti-infection therapy for patients postoperatively. Patients undergo esophagography or endoscopy 1–2 weeks after surgery to detect the potential presence of esophageal leaks. If no leaks are found, they can start to take a liquid diet.

## 3. Results and Discussion

### 3.1. Results

All 21 patients had a history of severe vomiting after drunkenness (11 cases, 52.4%), after eating a big meal (7 cases, 33.3%), and for other reasons (3 cases, 14.3%). Bloody vomitus was found in 3 cases (14.3%). All patients had suffered mild-to-severe chest or epigastric pain after vomiting, and other initial symptoms included shortness of breath (1 case, 4.8%), stuffy chest (2 cases, 9.6%), and nausea (4 cases, 19.0%). Moreover, 11 cases (52.4%) had sepsis symptoms during their hospitalization.

As shown in Table 1, positive results on X-ray were found in 9 cases (9/21, 42.9%). These results included widened mediastinum (4/9), hydropneumothorax (1/9), and mild-to-moderate pleural effusion (5/9). CT scans revealed positive results in 20 cases (20/21, 95.2%), including paraesophageal pneumomediastinum (18/20), esophageal tissue edema (19/20), hydropneumothorax (1/20), and pleural effusion (5/20). The one case with shortness of breath showed hydropneumothorax on X-ray and CT, and oral methylene blue was seen in closed chest drainage after thoracocentesis. The two cases with stuffy chest showed moderate pleural effusion on X-ray and CT, so they were definitely diagnosed by esophagography after receiving closed chest drainage. The one case with negative CT results was diagnosed by endoscopy.

In this study, 15 cases (71.4%) were firstly definitely diagnosed, and 6 cases (28.6%) were firstly misdiagnosed; the misdiagnoses included pneumonia, gastric ulcer, and intercostal neuralgia.

All patients were surgically treated (open surgery, but the last 3 cases underwent thoracoscopic surgery firstly for exploration) and then divided into three groups according to the time interval between symptom onset and surgical treatment: <24 h: 9 cases (42.8%); 24–48 h: 6 cases (28.6%); and >72 h: 6 cases (28.6%). All patients received hydration and broad-spectrum antibiotics preoperatively. 2 cases initially refused surgical treatment but then had to undergo surgery after 5 days of chest tube drainage because their sepsis deteriorated.

In the surgical findings, rupture of the middle esophagus was detected in 4 cases (19.0%) and of lower esophagus in 17 cases (81.0%). The average length of rupture was 2.2 cm (range, 0.8–7 cm). The ruptures were located on the left side of esophagus in 10 cases (47.6%) and on the right side in 11 cases (52.4%).

Primary esophageal repair was applied in all the patients in the <24 and 24–48 h groups (total of 15 cases) because the inflammation and empyema were not very serious. In the >72 h group, three of the six cases underwent primary repair, while the other three underwent debridement and drainage. Regarding the postoperative feeding methods, two cases received feeding via jejunostomy and 19 via a nasal feeding tube.

No patients died during the hospitalization in this study, as shown in Table 2. The rate of primary healing (no leakage occurred after primary esophageal repair) in the <24 h group was the highest (88.9%), as was the duration of hospitalization. The duration of hospitalization ranged from 12 to 66 days for all patients.

## 4. Discussion

Boerhaave's syndrome is always attributed to severe vomiting after consuming a large meal or an excessive amount of alcohol. Ruptures are most commonly located in the lower third of the esophagus and on the left side of the back wall of the esophagus, about 2–4 cm above the cardia [4]. This segment of the esophagus is a congenital weakness that lacks support and protection from surrounding organs and tissues. Patients usually continue oral intake after the onset of symptoms because of misdiagnosis, so the mortality associated with Boerhaave's syndrome is higher than that with other types of esophageal ruptures [5], the morbidity was reported as high as 40% [6].

The delayed diagnosis of Boerhaave's syndrome that results from its nonspecific symptoms can lead to serious mediastinitis and sepsis. It has been reported that the mortality associated with Boerhaave's syndrome is related to the time interval, with the highest mortality being as high as 50% [7]. CT is the most effective method for the early detection of pneumatosis around the esophagus. All of the cases in this study were scanned by CT, and the positive rate was 95.2%. Whether positive results can be shown by X-ray depends on three conditions: disease duration, site of rupture, and integrity of the mediastinal pleura. Patients may have no positive results on X-ray in the early period. The positive rate of X-ray in this study was only 42.9%, so we recommend that every patient with suspicious symptoms should undergo a CT scan in the emergency ward. In addition, although esophagography is a feasible and effective examination for suspected patients, it still has a false negative rate of 15–25%, which can be attributed to tissue edema or muscle spasms [8]. Endoscopy has the advantages of high sensitivity (100%) and specificity (80%) [9]. Other treatment methods such as hemoclip and stent placement can also be performed [10, 11]. However, endoscopy carries the risks of deteriorating the pneumomediastinum and enlarging the rupture, and the success rate of leakage sealing was still not satisfactory [12]. Moreover, these methods require experienced doctors with a high level of endoscopic skill, so we do not recommend endoscopy as a routine examination method. In this study, only one suspected case with negative results on CT underwent endoscopic examination, and a 0.7 cm rupture located in the lower esophagus was detected.

The treatment options for Boerhaave's syndrome include surgery and conservative treatment. Since the choice of treatment is closely related to the time interval, location, size of the rupture, and extent of chest infection and contamination, there is as of yet no definitive treatment. The therapeutic principles are as follows: limit diffusion of contaminations, adequate drainage, and efficient antibiotic treatment. According to current research and limited therapeutic experiences, surgery remains the most effective treatment for Boerhaave's syndrome. Without consideration of the time interval, surgery results in lower mortality than other treatments [5]; it has been reported that the mortality rate was 36% after operations delayed within 12 h, but if operations are delayed for ≥24 h, the mortality rate can increase as high as 64% [13]. Although the open approach was the common choice, successful treatment by thoracoscopic surgery was recently reported in a study with 12 cases [14].

For patients in the early period (<24 h), primary repair/drainage was widely accepted as the preferred treatment if tissues were viable [15], and the shorter the time interval, the lower the risk of postoperative leakage. The risk of leakage was 0% if the time interval for surgery was <6 h, 67% for 6–24 h, and 83% for >24 h [16]. The primary healing rate was 88.9% in the <24 h group, 66.7% in the 24–48 h group, and 0% in the >72 h group. Therefore, the footstone of successful treatment for Boerhaave's syndrome is to reach a definite diagnosis as early as possible. According to our experience, the following technical points for surgery should receive adequate attention: ① extend the rupture of the muscle layer to expose the entire length of the mucosal rupture and remove the necrotic muscle tissue; ② keep an appropriate needle pitch and margin and do not tie a tight knot to avoid cutting tissue; and ③ vascular tissue flaps can be used to reinforce the sutures, and successful treatment by omentum was also reported [17], but whether they can reduce the incidence of postoperative leakage still needs to be tested in further studies.

For patients in late period (>72 h), the ideal treatment strategy (conservation or surgery) is still under dispute. Conservative treatment mainly includes antibiotics and thoracentesis–tube drainage, and some studies [18, 19] have reported that several cases have been cured without surgery. However, there are no supporting tissues such as omentum majus in the chest, so inflammation can easily infiltrate throughout the mediastinum and cause severe sepsis in weak patients. Thoracentesis–tube drainage can lead to a good result in the early period, but thoracic separation commonly forms in the late period. As a result, adequate drainage can hardly be reached, and patients still have to undergo operations with debilitating sepsis. In this study, two patients who initially refused surgery had to undergo surgery after ineffectual conservative treatment, and thoracic inflammatory separations were confirmed in these operations. Similar results were also reported: nine of 21 cases receiving conservative treatment finally had to undergo surgical treatment [20]. For patients with severe sepsis, emergency debridement and drainage are still preferred, and whether to choose primary repair, delayed repair, or even partial esophagectomy depends on the operative exploration [5]. In order to facilitate postoperative enteral nutrition, jejunostomy will be applied. In this study, all patients recovered and were discharged from hospital after surgical treatment, regardless of the time interval. Although all of the patients in >72 h group had esophageal leaks postoperatively, they were well controlled because the leaks were much smaller; furthermore, the contaminations were all removed and adequate irrigation-drainage was performed surgically.

## 5. Conclusions

According to this study, surgery remains an effective method for treating Boerhaave's syndrome, and debridement, esophageal repair, lung restoration, adequate drainage, and sufficient nutrition support are all keys to success, so are the multidisciplinary treatments [21]. The findings demonstrate that the shorter the time internal, possibly the better the prognosis.

## Figures and Tables

**Table 1:** Auxiliary examination.

	n	Positive [n(%)]
Chest radiography	21	9 (42.9)
CT	21	20 (95.2)
Esophageal imaging/oral methylene blue	3	3 (100)
Gastroscope	1	1 (100)

**Table 2:** Surgical treatment and hospital stay.

	Primary repair [n (%)]	Primary healing [n (%)]	Hospital stay (days)
<24h (n=9)	9 (100)	8 (88.9)	18.1
<48h (n=6)	6 (100)	4 (66.7)	27.8
>72h (n=6)	3 (50)	0 (0)	51.2

## Data Availability

The data used to support the findings of this study are available from the first authors upon request.
